# Robust anticipation of continuous steering actions from electroencephalographic data during simulated driving

**DOI:** 10.1038/s41598-021-02750-w

**Published:** 2021-12-03

**Authors:** Giovanni M. Di Liberto, Michele Barsotti, Giovanni Vecchiato, Jonas Ambeck-Madsen, Maria Del Vecchio, Pietro Avanzini, Luca Ascari

**Affiliations:** 1 Henesis S.R.L., Strada Budellungo 2, 43123 Parma, PR Italy; 2 School of Computer Science and Statistics, Trinity College Dublin, Dublin 2, Ireland; 3 Institute of Neuroscience, National Research Council of Italy, Parma, Italy; 4 Toyota Motor Europe, Bourgetlaan 60, 1140 Brussels, Belgium

**Keywords:** Motor cortex, Cognitive control, Electroencephalography - EEG

## Abstract

Driving a car requires high cognitive demands, from sustained attention to perception and action planning. Recent research investigated the neural processes reflecting the planning of driving actions, aiming to better understand the factors leading to driving errors and to devise methodologies to anticipate and prevent such errors by monitoring the driver’s cognitive state and intention. While such anticipation was shown for *discrete* driving actions, such as emergency braking, there is no evidence for robust neural signatures of *continuous* action planning. This study aims to fill this gap by investigating continuous steering actions during a driving task in a car simulator with multimodal recordings of behavioural and electroencephalography (EEG) signals. System identification is used to assess whether robust neurophysiological signatures emerge before steering actions. Linear decoding models are then used to determine whether such cortical signals can predict continuous steering actions with progressively longer anticipation. Results point to significant EEG signatures of continuous action planning. Such neural signals show consistent dynamics across participants for anticipations up to 1 s, while individual-subject neural activity could reliably decode steering actions and predict future actions for anticipations up to 1.8 s. Finally, we use canonical correlation analysis to attempt disentangling brain and non-brain contributors to the EEG-based decoding. Our results suggest that low-frequency cortical dynamics are involved in the planning of steering actions and that EEG is sensitive to that neural activity. As a result, we propose a framework to investigate anticipatory neural activity in realistic continuous motor tasks.

## Introduction

Driving a car is a complex task requiring rapid planning and execution of motor actions. Shedding light on the neural underpinnings of driving actions requires a comprehensive set of physiological measurements capturing the various contributors to executive and motor functions. The muscular activity itself is a rich indicator of both current and upcoming actions (e.g., anticipatory muscle tensing^1^). A robust interaction was also measured between steering actions and eye-movements^2–4^. Crucially, brain processes leading to such motor actions have been associated with specific neurophysiological components^5–7^. Altogether, the combination of such direct and indirect indices of steering intention could lead to Brain-Computer Interfaces (BCIs) enabling the early detection of dangerous driving actions, warning the driver and potentially activating appropriate assistive driving tools before the action is taken. The present investigation aims to determine whether electroencephalography signals recorded non-invasively (EEG) reflect the driver’s steering intention while driving in a car simulator. Crucially, the study aims to disentangle cortical EEG signatures of steering from indirect non-cortical signals, thus providing us with a window into neural anticipatory processes.

Previous research on the neurophysiology of driving has focused on changes in brain state, showing that EEG signals reflect changes related to the driver’s attention, drowsiness, and workload^8–11^. Signals recorded with the same technology are also known to reflect voluntary movement. Both low-frequency EEG potentials (< 3 Hz), which are referred to as movement-related cortical potentials, and faster EEG activity, such as the sensorimotor rhythm^12^, have been related to the planning and execution of movements during both motor and imagery tasks^13–15^. The same signals have also been used to decode continuous three-dimensional hand movements^16–18^ to develop BCIs that could restore some level of mobility and control in individuals with full or partial loss of movement functions^13,19^.

Previous research on motor anticipation during driving focused on actions such as emergency braking, which are impulsive in nature and can be anticipated based on EEG and electromyography (EMG) signals^5^. Recent research found neurophysiological responses to emergency braking up to 800 ms before the executed event^7,20^. Part of the literature linked anticipatory EEG signals with the contingent negative variation (CNV), a central negative deflection that can last from about 300 ms to several seconds that was previously related to sensory-motor association and expectancy^21–23^. Measurements of the CNV from low-frequency EEG were used to decode the driver’s intention in realistic driving scenarios, showing that braking and accelerating actions can be decoded with anticipation of 320 ± 200 ms^6,24^. One crucial limitation is that such driving events have been treated as actions with discrete dynamics. As such, it remains unclear whether low-frequency EEG signals also reflect the anticipation of *continuous driving actions*. Furthermore, previous studies with motor planning tasks indicated that anticipatory signals are encoded in faster EEG dynamics related to sensorimotor neural activity (also identifies as mu rhythm^25,26^) spanning across the alpha- (8–13 Hz) and beta-bands (15-30 Hz)^27–29^.

Here, we investigate this question by testing whether EEG can anticipate continuous steering actions in a simplified yet realistic driving scenario on a driving simulator. The task involved driving on a mountain road track, with no road intersections nor traffic. We expect neurophysiological signals to reflect correlates of steering actions and anticipatory signals, such as the CNV. Crucially, the neural correlates of continuous steering while driving in a realistic scenario may reveal EEG signals beyond what was previously assessed for discrete events. In particular, we hypothesise EEG to reflect the steering actions with longer anticipations than previously seen for emergency braking and accelerating^6,7,20^, partly reflecting the planning that can often be performed well before executing the action. We assess this with a linear lagged regression approach (LLR), which explicitly isolates EEG correlates of steering with various anticipations. We consider EEG dynamics in the broad frequency-band 0.1–24 Hz, which includes EEG activity relevant to steering anticipation, including movement-related cortical potentials and sensorimotor rhythms^30^. Crucially, our analyses aim to explicitly isolate cortical EEG signature of steering intention from indirect, non-cortical contributors reflecting eye-movements and anticipatory muscle activation. In doing so, we demonstrate a framework for the anticipation of continuous steering actions in a simulated driving scenarios sensitive and capable of discerning cortical and non-cortical signatures of driving intention.

## Results

**Figure 1:**
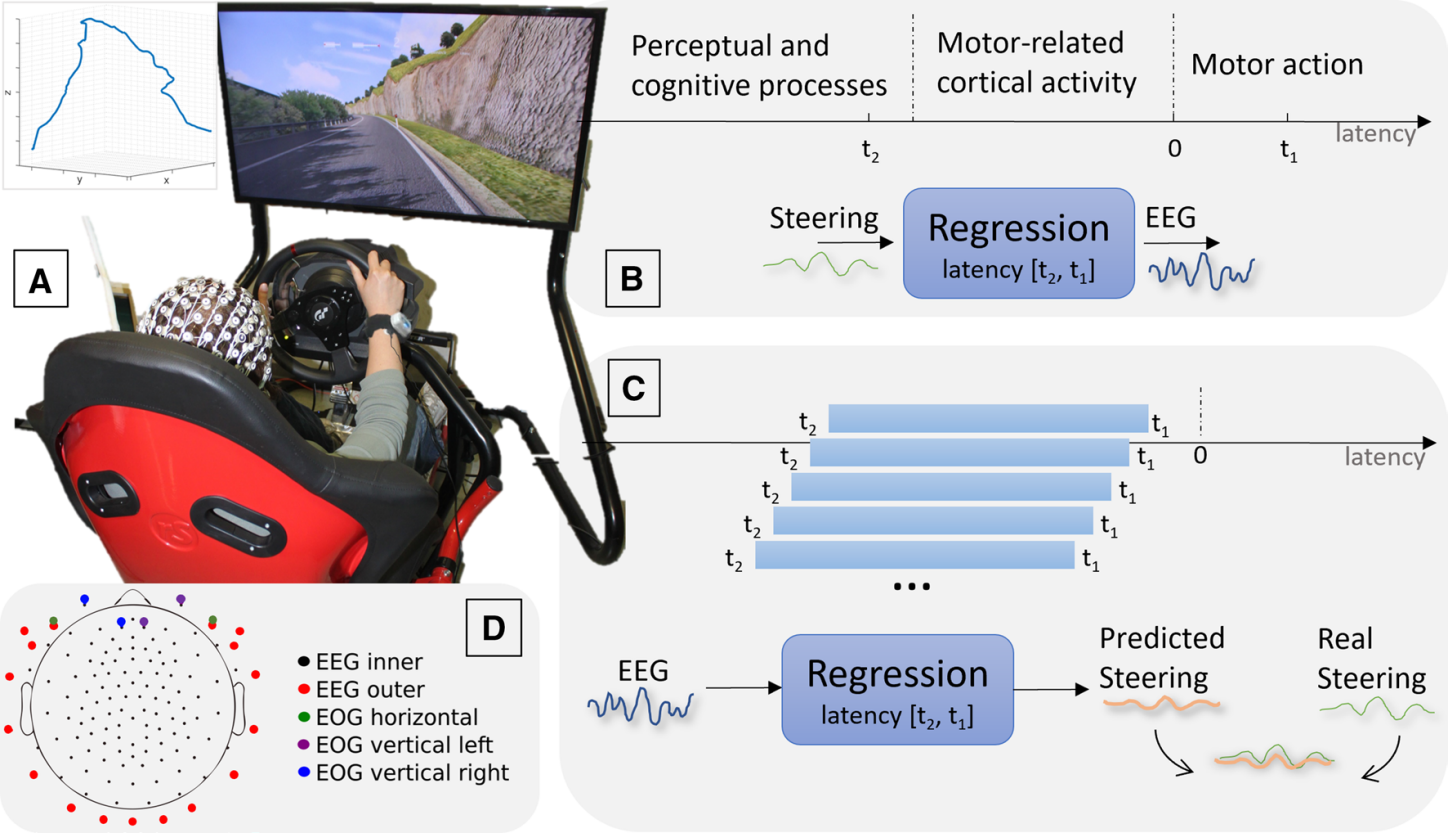
Experimental setup and analysis framework. (**A**) Experimental Setup. Subject performing the experiment inside the racing simulator (Assetto Corsa). Top-left a 3D plot of the track is shown. (**B**) Encoding Model. Lagged linear regression (LLR) encoding models were fit to estimate EEG dynamics preceding and following steering actions with a steering-EEG latency window from –1.5 s (t2) to 0.5 s (t1). (**C**) Decoding model. LLR decoding models were fit to predict the steering position from preceding EEG data at latencies between t2 and t1, with progressive longer anticipations and a fixed window-size of 1.5 s. The quality of the predicted steering signal was assessed with Pearson’s correlation. (**D**) EEG-layout. EEG 128-channels location divided by colours (black thin dots are the inner electrodes used for LLR).

EEG signals were recorded from thirty-one volunteers as they performed a simulated driving task on a racing simulator. Data from five participants were excluded due to a synchronisation issue (see Methods). The steering wheel’s position was recorded into a continuous *steering signal* (S) and compared with the EEG (0.1–24 Hz). That relationship was studied with a lagged linear regression analysis (LLR) to assess the EEG encoding of S (Fig. 1). First, *encoding LLR models* were fitted with a large temporal window to describe the forward linear relationship from S to the EEG signal. This approach was used to test whether significant neurophysiological correlates of S emerge that anticipate and follow the action. Then, the continuous steering signal was reconstructed from the EEG signal by using *decoding LLR models* with progressively increasing anticipation, which describe the backward relationship from the EEG signal to S.

### Robust EEG components anticipate steering actions

**Figure 2:**
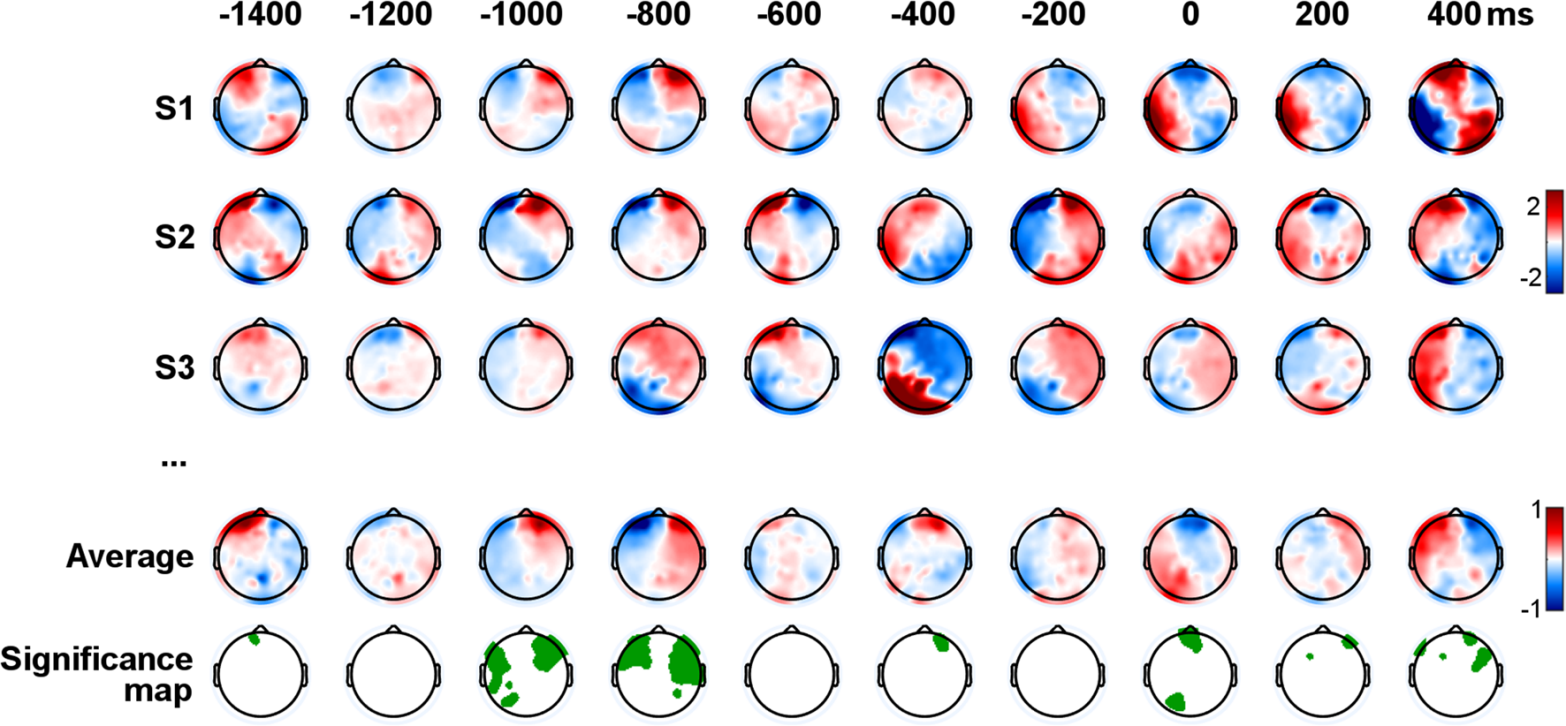
Significant EEG signals anticipate steering actions. LLR encoding models describing the EEG dynamics that precede and follow steering actions. Colours indicate LLR regression weights for individual participants and the average model. The bottom row shows electrodes with weights significantly different from zero (*p* < 0.05, FDR-corrected Wilcoxon test across participants).

The forward relationship between S and the EEG signal was assessed with LLR, which identifies an optimal linear transformation from S to the EEG for each channel. The mapping describes the linear relationship between the continuous steering and the EEG. Specifically, the LLR model estimates the EEG dynamics that precede (negative lags) and follow (positive lags) a steering action by taking into account pre-defined temporal shifts between S and the EEG. Such temporal shifts, which we referred to as the *latency window* of the model, were set to allow us to assess EEG responses up to 1.5 s of anticipation and 0.5 s of delay compared to S (see Methods). Significant EEG prediction correlations scores were measured with a tenfold cross-validation procedure at the individual subject-level. This result is depicted in Fig. 2, where colours indicate LLR weights in particular scalp areas and latencies. The LLR weights are commonly referred to as the Temporal Response Function (TRF^31,32^), where large weights (either positive or negative) reflect a strong EEG signal that either leads to (negative lags) or follows (positive lags) a steering action. Significant TRF components were measured on the average LLR model across all 26 participants (Fig. 2, bottom row; *p* < 0.05, Wilcoxon signed-rank test with FDR-correction for multiple comparisons across latencies), indicating that, despite the expected large inter-subject variability, the LLR analysis found TRF components that were consistent across participants that both preceded and followed steering actions.

### EEG decoding of steering actions

The encoding model results indicated robust anticipatory EEG components that precede steering actions for up to 800 ms. We then used LLR to assess whether upcoming steering actions can be predicted based on the preceding EEG signal. To do so, we fit LLR decoders with 1.5-s-long latency windows with progressively longer anticipation, from 0 to 3 s. As a control analysis, we have also performed the decoding for long anticipations of 6 and 12 s, where we did not expect to find a significant relationship between S and the EEG signal. The quality of the decoding was assessed through the Pearson’s correlation index between the predicted (Ŝ) and the actual (S) steering signals via a tenfold cross validation procedure (see Methods).

**Figure 3:**
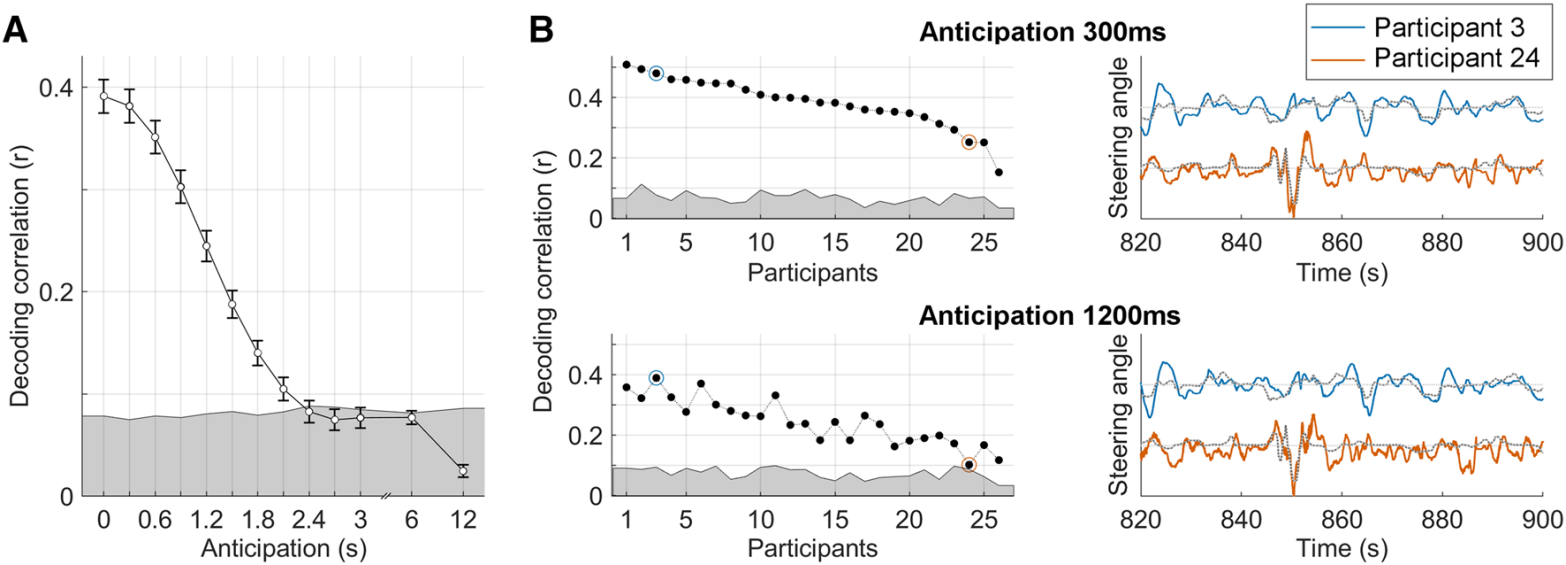
EEG decoding of continuous steering actions. (**A**) Backward TRF models were used to decode the steering signal from unseen EEG data with cross-validation. Steering reconstruction correlations are reported for TRF models with various anticipations (with a fixed TRF window-size of 1.5 s). Error-bars indicate the SE across participants. The grey area indicates the chance level, which was the 99th percentile of a null distribution calculated by re-running the regression analysis 100 times on shuffled steering/EEG data (calculated across all participants and shuffles). (**B**) (Left) Individual-subject steering reconstruction correlations for selected anticipation windows. Participants were sorted according to the results with anticipation 300 ms. (Right) Selected portion of the steering signal (grey lines) and the corresponding steering reconstructions for two selected participants. Decoding results for those participants are also indicated in panel B with the same colours.

To test whether S can be predicted from EEG, we re-run the lagged regression for various latency windows with fixed window size (1.5 s) and progressively increasing anticipation (Fig. 3A). As we had hypothesised, anticipation had a main effect on the decoding correlation (one-way repeated measures ANOVA, *F*(12,300) = 297.0, *p* = 2.2*10^–33^). Significant steering predictions were obtained for all participants for anticipations up to 1.2 s, indicating a robust encoding of S in the EEG signal (Fig. 3B). As expected, the EEG decoding of S progressively degraded for longer anticipations. Specifically, significant results were found for 25 out of 26 participants, 22/26, 18/26, 13/26, 13/26, 12/26, 9/26, and 1/26 participants for anticipations for up to 1.5, 1.8, 2.1, 2.4, 3, 6 and 12 s respectively (two-tailed permutation tests with FDR-correction for multiple comparisons). Note that we have also re-run the same analysis for low-frequency EEG in the 0.1-3 Hz band and the results did not change, while the decoding result was non-significant for EEG filtered between 3 and 24 Hz, thus indicating that low-frequency EEG dynamics drive most of the LLR decoding.

### Isolating the cortical signals contributing to the steering decoding

**Figure 4:**
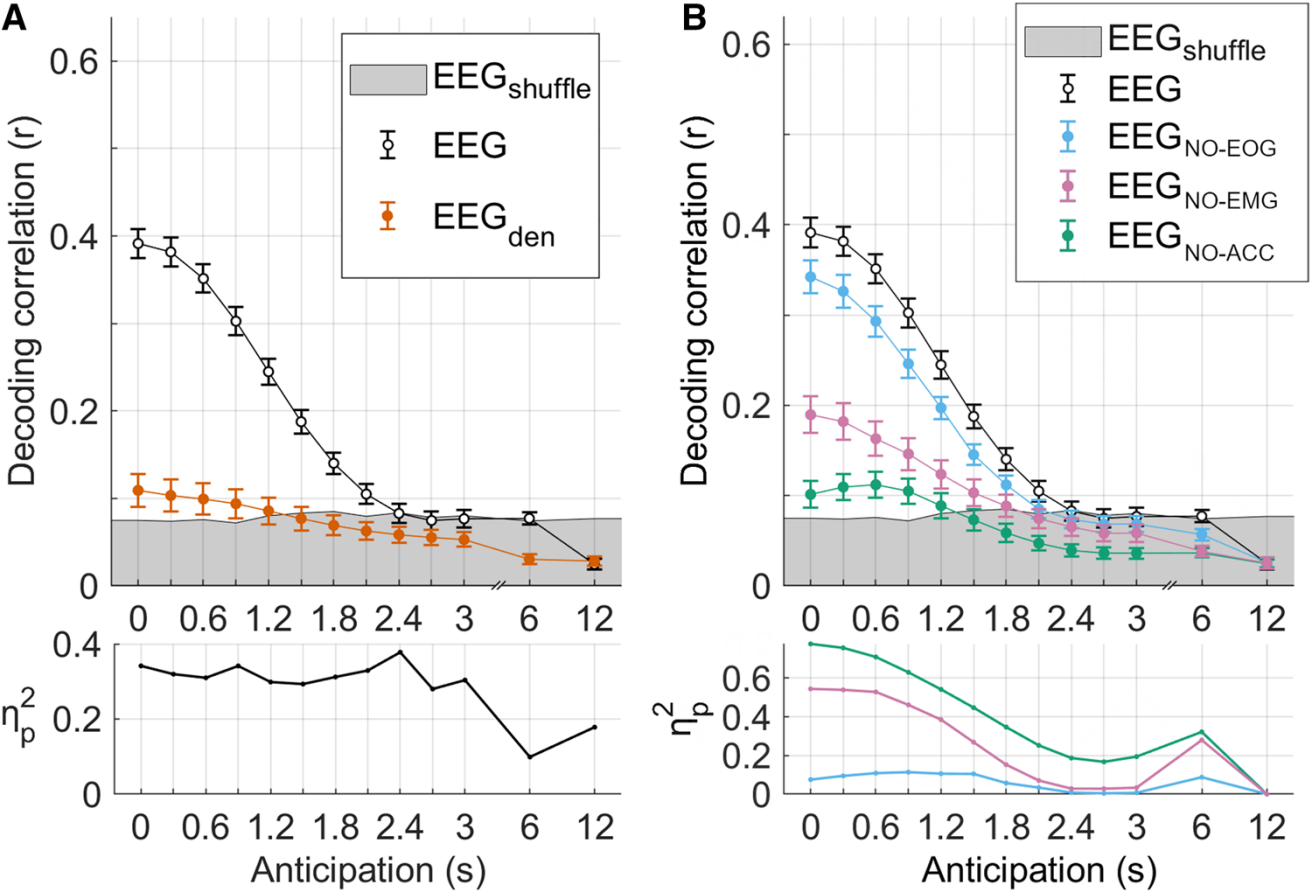
Disentangling cortical signals contribution to steering decoding. (**A**) Steering decoding correlations using EEG data with progressively longer anticipation, with the LLR window set to 1.5 s. Results indicate decoding correlations when using the EEG before and after removing motion-related components (EEG and EEGden respectively). Error-bars indicate the SD across participants. The grey shaded area indicates the baseline prediction correlation (95^th^ percentile of the null distribution). The plot on the bottom reports the effect-size for EEGden > baseline. (**B**) The steering signal was prediction by removing selectively each potential non-brain signal. The LLR window was set at 1.5 s. Error-bars indicate the SD across participants. The grey shaded area indicates the baseline prediction correlation (95th percentile of the null distribution). The plot on the bottom reports the effect-size for EEG > EEG_NO-SIGNAL_ for each signal of interest.

The remarkable decoding scores measured in Fig. 3 are in line with what would be desirable for a potential real-world BCI application of this work. Indeed, one challenge is to first investigate what the factors contributing to the decoding are, allowing us to determine if properties that would be present in real-world scenarios are measurable with this approach. To this end, we tested if the decoding was driven by cortical activity rather than potential confounding factors present in the EEG data, such as motion-related and eye-movement EEG signals. To assess the impact of motion-related signals, accelerometric data were recorded during the experiment from the neck, chest, and wrists of the participants, and EMG data were also collected from the deltoid and the extensor digitorum muscles (left and right). A denoising procedure based on canonical correlation analysis (CCA) was then applied to isolate the EEG components strongly correlated with EMG, accelerometric, and eye-movement data and assessing their impact on the decoding analysis by excluding them from the EEG signal. The availability of signals reflecting the temporal dynamics of potential confounders made CCA an optimal choice for the EEG denoising, as the procedure explicitly isolates components that are maximally correlated to the known noise temporal series (differently from, for example, the widely used ICA method which is a blind-source-separation method; see Supplementary Figure 1 for a visual representation of CCA-based denoising). The resulting denoised EEG signal is referred to as EEGden (see Methods). The steering signal was significantly decoded from EEGden (Fig. 4A), showing a main effect of anticipation on the decoding correlation (one-way repeated measures ANOVA, *F*(12,300) = 11.0, *p* = 1.0*10^–4^). Significant results were found at the individual subject level for at least 50% of the participants for anticipation up to 1.8 s (two-tailed permutation tests with FDR-correction for multiple comparisons; the bottom panels in Fig. 4 also report the effect size for each anticipation value). For longer anticipations (i.e., 2.1, 2.4, 2,7, 3, 6 and 12 s), the percentage of subjects who achieved significant results substantially decreased (only 12 out of 26 participants, 10/26, 9/26, 6/26, 4/26, and 1/26 participants showed significant results, respectively).

To isolate the effect of potential non-brain EEG signals on the steering decoding, we have performed an additional analysis where we attempt to selectively remove EEG components that were correlated with the steering action but are not directly reflecting brain activity. Note that these confounders are useful signals when the goal is to achieve the best prediction of future steering actions. Nevertheless, we were interested in separating brain and non-brain contributions to the decoding to clarify what exactly is driving our steering decoding results. Figure 4B shows the decoding correlation for the EEG and its “denoised” version, from which contributions of EOG, accelerometers and EMG have been removed. A two-way repeated measures ANOVA was conducted using as factors *models* (4 levels) and *anticipations* (13 levels). Strong significant main effects of models (*F*(3,75) = 49.0,* p* = 2.7*10^–13^) and anticipations factor (*F*(12,300) = 199.0,* p* = 4.8*10^–29^) were found. Also, a significant interaction effect (*F*(12,300) = 199.0,* p* = 4.8*10^–29^) was found reflecting the progressive decreasing decoding correlation with increasing anticipation. It can be noted that, for all anticipations, the stronger contribution is the one relative to the accelerometers whereas the minor contribution is relative to the EOG signals.

These results rely on the assumption that the previous CCA analysis did, in fact, remove all the relevant non-brain components, rather than just part of them. For this reason, we have performed an additional analysis to isolate the cortical signals contributing to the steering decoding. In this new analysis, instead of removing accelerometric and EMG signals from the EEG data, we have combined EEG, EMG, accelerometric data, and eye-movement components to decode S (Supplementary Figure 2). We have then assessed the impact of the EEG on the decoding by repeating the same analysis after time-reversing the EEG signal, which was therefore meaningless for the decoding. This analysis showed an impact of the EEG on the decoding that increases with anticipation, peaking at 3 s (two-way repeated measures ANOVA: main effect of model, *F*(1,25) = 46.1, *p* = 4.1*10^–7^; main effect of anticipation, *F*(12,300) = 311.6, *p* = 3.1*10^–31^; model per anticipation interaction, *F*(12,300) = 8.4, *p* = 3.1*10^–4^). Note that the strength of the effect in Fig. 4B quantifies the EEG information relevant to the decoding that is unrelated to motion artifacts. As such, the increasing pattern for anticipations up to 3 s does not mean that the EEG signal more strongly encodes S with longer anticipation, but instead that the encoding becomes progressively more complementary to motion-related signals.

## Discussion

This study demonstrates that EEG signals recorded during simulated driving *encode* steering actions for up to 3 s before the execution onset. Crucially, the decoding analysis showed that the EEG signals could reliably predict continuous steering actions with varying anticipation. The implications of this finding are multifaceted. This work provides us with a view into the neurophysiological underpinnings reflecting a driver’s intention for continuous steering actions with a real-life task. Furthermore, brain and non-brain EEG components reflecting such anticipation were isolated and found consistent within each participant across the task duration, despite the steering actions having various duration and rapidity. Finally, the objective indices derived from our analyses could potentially lead to various applications, ranging from the anticipation of hazardous action to monitoring cognition while driving.

The finding that EEG signals reflect the driver’s intentions on continuous motor actions bridges the gap between work on real-time continuous movement decoding^16–18^ and discrete action anticipation^6,7,20^. Our findings indicate that EEG signals (0.1–24 Hz) encode the driver’s intention to steer during a realistic driving task, resulting in consistent EEG activity with lateral premotor scalp maps around 1000 ms before the action onset. Furthermore, the LLR analyses were driven by low-frequency EEG signals < 3 Hz predicting the steering onset up to 2.4 s before the action (as decoding correlations do not change when using EEG data low-pass filtered at 3 or 24 Hz).

Interestingly, the topographical patterns of significance resulting from the LLR forward models could be reminiscent of the spatial scalp distribution of mu rhythm. Mostly arising from the motor and premotor regions^26,33,34^, the mu rhythm is known to exhibit somatotopically organised desynchronisation of 10- and 20-Hz components during execution, observation, and imagination of actions^25,35,36^. The topographical parallelism between anticipatory EEG features and cortical motor rhythms is in line with the notion that the motor system performs several functions other than control of body movements, such as sensorimotor transformation, action understanding, decision processing regarding execution and initiation of action, preparation, and planning of complex movements^37,38^. On the same dataset, we already showed how steering actions elicit a desynchronisation of 10- and 20-Hz components across sensorimotor regions^39^. It is then tempting to propose mu rhythm as the basic EEG feature to predict upcoming actions.

In partial disagreement with this conclusion, the EEG analysis performed with the LLR backward models confined most of the capacities of steering actions decoding to low-frequency EEG components, while no significant contribution was brought by the frequency bands specific for the sensorimotor rhythms. These findings are also in line with previous studies showing that EEG signals under 3 Hz can decode arm and hand movement as well as an individual’s intention to move^40^. The low-frequency neurophysiological responses measured in those tasks were often associated with the CNV component, which is described as a centrally, midline-distributed slow potential (within the 1–3 Hz range of the delta-band) emerging in the interval between the presentation of a cue and the next target^41^. We cannot exclude that a CNV-like activity is present in the EEG signal and plays a role in the decoding, but that the topographical patterns reflect more dominant EEG variations related to motor planning and action anticipations^28^. In summary, our findings exhibited a pattern intermediate between sensorimotor rhythms and low-frequency potentials, where low-frequency EEG resulted critical for the steering decoding analysis, but topographical patterns that are not typical of the CNV component were measured instead (Fig. 2).

Multiple reasons could underlie this partial mismatch. Slow motor potentials preceding movement initiation are usually observed in laboratory settings allowing a precise timing control of contextual cues and the corresponding target signals. Instead, our realistic experimental design did not provide us with the precise timing of such contextual cues (e.g., the right turn at the end of the street), which may have been processed by the users with large advance relative to the time of the motor action. As such, in addition to making the computation of prototypical ERP components suboptimal, the particular experimental design possibly presented neural motor activity with different dynamics compared to previous studies. Besides, one should account that steering represents a continuous behaviour, thus eliciting evoked responses that should be studied with methods that account for such continuous dynamics which, however, may not be directly comparable with the more traditional ERP averaging method. In a speculative perspective, we could propose that our pattern of findings reflect the simultaneous recruitment of different motor circuitries. A lateral sensorimotor network would reflect the planning of steering actions that, to be performed, would need the control and release of the motor command by medial frontal areas. Future studies might address this point, possibly manipulating the experimental stimuli and recording behaviour together with neural signals providing mechanistic insights into the cortical spatiotemporal dynamics.

This work provides us with a methodology for monitoring driving actions. Here, we go beyond the study of isolated discrete actions, providing instead evidence into the neural processing of continuous driving actions. As expected, continuous actions showed neural correlates with substantially longer anticipation than previously seen for emergency braking^7,20^ (frontal alpha and theta power synchronization occurring from 600ms^7^ to 800ms^20^ before the onset of the executed braking event, respectively), as the latter is rapid by definition, while continuous steering actions involve a longer planning process. One further consideration is that the driver’s ability to perform early planning of motor actions changes with factors such as fatigue and tiredness. As such, the possibility of measuring EEG correlates of steering anticipation may then index the driver's cognitive state and, specifically, enable the continuous monitoring of critical factors for driving, such as attention and tiredness. In this sense, our finding that driving actions can be accurately decoded with substantial anticipation could contribute to systems for the monitoring and early detection of potentially dangerous actions while driving. The CCA-LLR methodology proposed here provides a solution to disentangle the various EEG contributors that are relevant to steering, which we expect to be effective even when recording EEG data in an actual car during driving. One more challenging question, which was beyond the scope of this study, is how the present finding extends to scenarios involving external agents, such as other cars or pedestrians. In those cases, in fact, such external agents likely impact the steering actions and the underlying cognitive processes. The isolation of anticipatory neural indices of driving actions would then likely benefit from methodologies modelling the movement of such external agents, similarly to the predictive processes occurring in the driver’s brain. Further studies should investigate this challenge to clarify whether the present finding generalise to even more complex, real-world scenarios.

One challenge to neurophysiological investigations with realistic driving tasks is the presence of large non-brain EEG signals. The portions of the signal reflecting components that are unrelated to the target of the investigation (e.g., the steering signal) can reduce the SNR of the EEG signal. Nevertheless, their independence from the target signal allows algorithms such as LLR to effectively isolate different signals contributing to the measured EEG potentials. The use of CCA-denoising provides instead a solution for removing a different type of non-brain related EEG portion that emerges during simulated driving task and is potentially more challenging. Namely, EEG components reflecting motion artifacts that are correlated with the motor action itself. Here, we proposed using CCA to denoise of the EEG data from such artifacts, while LLR allowed us to disentangle EEG activity that preceded and followed the action. This combination of CCA-denoising and LLR was particularly effective when investigating which EEG components most dominantly contribute to the decoding result. In this regard, the result in Fig. 4A demonstrates that the EEG signal could reliably predict steering actions even after removing EEG components related to motion and eye-movement. While our analysis may not be sufficient to fully isolate cortical EEG signals from non-brain signals, we contend that it accounts for the most dominant components whose effect reported individually in Fig. 4B. This finding is even more relevant considering that the adopted denoising procedure (CCA denoising) is highly conservative, as it attempts to remove all EEG portions related to the nuisance regressors (eye-movement, accelerometers, EMG). In other words, the procedure may also remove portions of the EEG reflecting cortical signals correlated at zero-latency with the regressors, thus making this result even more convincing. Future studies should also consider the possibility that other relevant non-brain sources existed which were not taken into account by our analysis because uncorrelated to the nuisance regressors (for example, there may be ocular components that were not captured by the eye-movement signal used in this study such as eye rolling during fixations). However, it is worth of note that to optimise the removal of the non-brain components while preserving the brain signals, CCA targeted only instantaneous correlations (differently from LLR, which accounted for time delays), making it an optimal procedure for the removal of non-brain components instantaneously related with the nuisance regressors (EOG, accelerometers, and EMG), while brain signals were expected to be anticipated and delayed compared with the corresponding actions, but not simultaneous.

While motion-related EEG signals are present both in tasks involving continuous and discrete motor actions, the study of intentions during continuous actions presents new challenges compared with previous work on discrete actions (e.g., steering and emergency braking). Here, one such challenge was the positive serial correlation of steering signals, meaning that the proximal history of steering positions can somewhat predict the immediately following steering position. While this can work in favour of BCI development, as it can improve the decoding scores, studying the neurophysiological signature of the driver’s intention benefits from disentangling them from such serial correlation effects. The analysis in Supplementary Figure 2 represents a clear demonstration of such a separation. Specifically, we could show that low-frequency EEG signals encode steering intention with long anticipations (up to 3 s), which could not be measured instead from accelerometric, EOG, and EMG signals. The present study showed that the CCA-LLR analysis is effective in detecting EEG signals that anticipate steering actions in a continuous driving simulation task. Crucially, this approach could discern the relative contribution of behavioural and electrophysiological components to the steering decoding. Although a large contribution was measured for non-brain related signals, such as ocular and muscular components, we also provided evidence that part of the decoding was driven by cortical signals, suggesting that EEG signals reflect anticipatory neural signatures of continuous steering actions. These results pave the way for the utilisation of multimodal recordings in driving settings for the online prediction of salient actions.

## Methods

### Participants

Thirty-one subjects (8 female; median = 23 years of age; min = 19 years; max = 41 years; all of them were right-handed) participated in this experiment. All participants declared that they were right-handed and had normal or corrected to normal vision. All of them held an Italian driving license and none of them had prior driving experience on professional racetracks. Participants gave written informed consent to participate in the study. The study was undertaken in accordance with the Declaration of Helsinki and approval for the study was obtained from the local ethical committee (Comitato Etico Unico per la Provincia di Parma). Data from twenty-six participants were analysed in this study because of data synchronisation issues with the other subjects (7 female, median = 23 years of age; min = 19 year; max = 27).

### EEG experiment

EEG data were recorded as participants performed a driving task on a driving simulator composed of: RSeat RS1 Assetto Corsa Special Edition (seat); Thrustmaster T500 RS (steering wheel and pedals); Samsung 40″ 5300 class LED TV positioned 1 m from the participant’s seat (vertical field view = 27.4°, horizontal field view = 46.8°); and Assetto Corsa software (Kunos Simulazioni) (Fig. 1). The car selected for the experiment was an Alfa Romeo Mito with automatic transmission and 100% stability. Before the experiment, participants were asked to drive on the Monte Erice track (6.9 km, https://assettocorsa.club/mods/tracks/monte-erice.html) for a single lap. Next, EEG was recorded as participants performed a single lap on the Coste Loop track (9.7 km, https://assettocorsa.club/mods/tracks/coste-road.html), with the only constraint of maintaining the right lane. Participants took 21.4 ± 3.7 min to complete the lap. No other vehicle was present on the tracks. Participants were asked to drive as if they would in a realistic scenario.

During the experiment, EEG data from 128-scalp positions were recorded with a Geodesic EEG system (Electrical Geodesics Inc, Eugene, OR, USA) and the HydroCel Geodesic Sensor Net. EEG data were obtained with sensor-skin impedance maintained below 50 kΩ using high-input impedance amplifiers (Net Amps300). Signals were digitised at a sampling rate of 500 Hz (0.1 Hz high-pass filter) and recorded with a vertex reference with impedance below 10 kΩ. Eye-movement electrical signals were extracted by means of two approaches: directly using the bipolar EOG channels (green, purple and blue electrodes pairs in Fig. 1D) and by visual identifying and extracting blink- and saccades-related components in the ICA analysis (see Supplementary Figure 1 3rd row, 2nd-3rd columns for a visual comparison of signal extracted by the two approaches).

Bipolar EMG data (ENOBIO, sample frequency 500 Hz) were recorded from 4 positions (deltoids, left and right; extensor digitorum muscle, left and right). Accelerometric data were recorded using inertial units (mbientlab IMU, 50 Hz) placed on the participants’ neck, chest, and wrists (left and right). Telemetry and behavioural data from the driving simulator (steering, gas, velocity, etc.…) were recorded at 250 Hz. Lab-streaming-layer (LSL^42^) data acquisition and control framework^43^ was used for recording data during the experiment. Moreover, precise synchronisation between EEG and the other LSL streams was achieved by means of two photodiodes placed in front of a screen that switched periodically from black to white every 7 s. Each visual switch was captured by two luminance sensors that sent a synchronisation trigger to the EEG system through LSL.

### Data pre-processing

EEG data were pre-processed and analysed in the MATLAB (R2019b, The MathWorks) with custom code using functions from the open-source libraries NoiseTools (http://audition.ens.fr/adc/NoiseTools/), mTRF-Toolbox (https://github.com/mickcrosse/mTRF-Toolbox), and EEGLAB (v2019.0). EEG data were filtered between 0.1 and 24 Hz with a zero-phase band-pass Butterworth filter with order 4 + 4, and down-sampled to 100 Hz. Seventeen outer electrodes were removed as they were placed over areas prone to muscular artifacts. Data were re-referenced to the average of all 111 remaining channels. Electrodes with variance larger than three times the mean variance across all electrodes were labelled as noisy and excluded from the analyses that follow^44^.

EMG data were analysed to remove likely muscular noise from the EEG signal. To this end, the EMG data was preprocessed in two different ways: a) signals were isolated from the EMG sensors by applying the same preprocessing applied to the EEG data; b) the linear envelope was extracted by band pass filtering (20–250 Hz) and computing the root mean square value of the signal within sliding windows of 250 ms. As a result, two EMG preprocessed signals were extracted for each pair of EMG sensors, providing us with a total of 4 vectors. The 6-dimensional accelerometric vector (acceleration and angular velocity along the three axes) was z-scored. The steering position was collected as part of the Telemetry data and did not undergo any preprocessing, as it indicated already the angular value of the steering wheel using a univariate vector.

### CCA denoising

EEG data were recorded during a realistic driving task that involved continuous motor actions. For this reason, EEG signals were subjected to contamination by noise, such as muscular noise and movement artifacts (head movements). A canonical correlation analysis (CCA^45^ was performed to reduce such artifacts by isolating and removing EEG components highly correlated with the pre-processed EMG and accelerometric data. Specifically, CCA was run between the EEG data (111 channels) and a *noise matrix* consisting of the concatenation of deltoid EMG, neck and wrist accelerometers, and EOG. To do so, the CCA function in the toolbox *NoiseTools* was utilised (http://audition.ens.fr/adc/NoiseTools/^46^). Given two sets of multichannel data X (the noise signals) and Y (the EEG signal) of size T x J1 and T x J2, where T is the number of time samples, CCA finds the best linear transform W1 (size J1 x J0, where J0 < min(J1,J2) to apply to the first to maximise its projection on the second, and the best linear transform W2 (size J2 x J0) to apply to the second to maximise its projection on the first. Specifically, W1 and W2 maximise the correlation between pairs of columns of XW1 and YW2, while making the columns of each transformed data matrix mutually uncorrelated. The first pair of canonical components (CC) is the linear combination of X and Y with highest possible correlation. The next pair of CCs are the most highly correlated combinations orthogonal to the first, and so-on. Note that CCA is a linear technique, but it can be extended to characterize non-linear and convolutional relations by including appropriate transforms of the data before applying CCA.

In the present study, J2 was 111 as it corresponded to the number of inner EEG channels. The dimensionality of X, representing the temporal series reflecting potential EEG noise, depended on various factors: 4 EMG signals preprocessed like the EEG; 4 EMG envelope signals (see previous sub-sections); 6 signals for each of the two accelerometers; and 3 bipolar signals capturing the eye-movement signals. In addition to these components, we included in X signals containing white noise so that J1 = J2, which made the CCA mapping a reversible data mapping. Otherwise, a denoising performed with J1 < J2 could change Y even for unrelated X, due to data compression.

This procedure allowed us to rotate the EEG data into a space where the first component is the one maximally correlated with the noise matrix. We then back-projected the neural data from CCA to the EEG domain after removing the canonical components that were largely correlated with the EEG-noise. Specifically, we excluded the first k components with EEG-noise correlation higher than the 90th percentile of the distribution of correlations across all CCA components within the given CCA. Note that the choice of using the 90^th^ percentile was arbitrary and that the overall considerations of this study do not change for other reasonable choices of that parameter. The EEG data were then back-projected into the original space, providing us with the denoised EEG data matrix (EEG_den_).

### Lagged linear regression analysis (LLR)

The relationship between EEG data and steering actions was assessed with an LLR analysis. LLR consists of a ridge regression that estimates the optimal linear mapping between an input (*x*) and an output signal (*y*). This mapping is performed considering that each value in the output signal *y*_*t0*_ may depend on the value of *x* at multiple time-latencies or, equivalently, the value of *x* at a particular time t_1_ may impact *y* for a given duration. LLR allows investigating the interaction of x and y across a given time-lag window, which should be large enough to include the latencies where that interaction is hypothesised to be most prominent. LLR includes a regularisation procedure, which practically results in the temporal smoothing of the regression weights, increasing the generalisation of the model. The optimal regularisation parameter (lambda) is identified by means of an exhaustive search within a given logarithmically spaced interval, here set between 10^–3^ and 10^9^.

The LLR analysis was conducted by using the mTRF-Toolbox (version 1.5; https://github.com/mickcrosse/mTRF-Toolbox)31. First, forward LLR models were estimated to assess the EEG dynamics preceding and following the steering action. The resulting regression weights, referred to as the TRF, were studied in terms of their spatial and temporal dynamics, similar to an ERP analysis (Fig. 2). The TRFs obtained from individual participants were averaged to obtain the grand average TRF waveform. This analysis was performed by considering the time-lag window from -1.5 to 0.5 s, which was considered sufficiently large to capture most of the EEG signals of interest. A ten-fold leave-one-out cross-validation was used to control overfitting. Specifically, the mapping between X and Y was estimated on each unseen fold with an LLR model fit on all other folds. The procedure was then iterated for all combinations. Pearson’s correlation values (*r*) quantifying the decoding quality were then obtained for individual participants by averaging *r*-values across the ten test folds.

Second, backward LLR models were fit to decode the steering signal from the EEG data. The decoding was assessed by considering various 1.5 s-long latency windows preceding the motor action with progressively increasing anticipation (0, 0.3, 0.6, 0.9, 1.2, 1.5, 1.8, 2.1, 2.4, 2.7, 3.0, 6.0, 12.0 s of anticipation). Specifically, subject-specific models were fit on a training portion of the data to determine the optimal LLR mapping for the decoding of steering actions. For example, a model was fit for the 1.2 s anticipation based on the first part of the driving experiment by using an LLR latency window from 2.7 s to 1.2 s of anticipation (i.e. [-2.7 s,-1.2 s]). In other words, let’s consider a time t1. That LLR model uses the EEG signal recorded [t1-2.7 s, t1-1.2 s] to predict the steering action at time t1. Such a prediction is performed for each time sample *t*_*i*_ on unseen portions of the data. A ten-fold leave-one-out cross-validation procedure was used to perform this model fit and decoding operations while controlling for overfitting. The quality of the decoding was assessed by comparing the predicted and the actual steering signals with Pearson’s correlation. Correlation values were averaged across folds, resulting in one value for each subject and anticipation^31^.

### Statistical analysis

Statistical analyses were performed using one- and two-way repeated measures ANOVA, with latency and decoding model as independent variables and decoding correlation as the dependent variable. The two-way repeated measures ANOVA accounted for interactions between its factors. Greenhouse–Geisser corrections were made if Mauchly's test of sphericity was not met. Post hoc comparisons were assessed with Wilcoxon signed-rank tests. Correction for multiple comparisons was applied where necessary via the false discovery rate (FDR) approach^47^. ANOVA results were reported using the convention *F*(*df*, *dferror*). Partial eta squared (ηp^2^) was used as a measure of effect size. Baseline decoding correlation values for the LLR analyses were calculated by re-running the LLR cross-validation procedure 100 times, each time after randomising the fold identities by shuffling the order of the EEG fold labels. In other words, the LLR procedure was run after pairing each steering signal fold *i* with a random EEG fold *j*. The baseline to values were set to the 95th percentile of the distribution of decoding correlations values obtained on the randomised data.

## Supplementary Information

Supplementary Information.
