# Supplementary material for: Robust anticipation of continuous steering actions from electroencephalographic data during simulated driving
Source: Sci Rep. 2021 Dec 3;11:23383. doi: 10.1038/s41598-021-02750-w (PMC8642531; doi:10.1038/s41598-021-02750-w)
Supplement: Supplementary file 1 — Supplementary Information. [file 41598_2021_2750_MOESM1_ESM.pdf]

Robust anticipation of continuous steering actions from electroencephalographic data during simulated driving

Giovanni M. Di Liberto[1,2,*], Michele Barsotti[1,*], Giovanni Vecchiato[3], Jonas Ambeck-Madsen[4], Maria Del Vecchio[3], Pietro Avanzini[3], Luca Ascari[1]

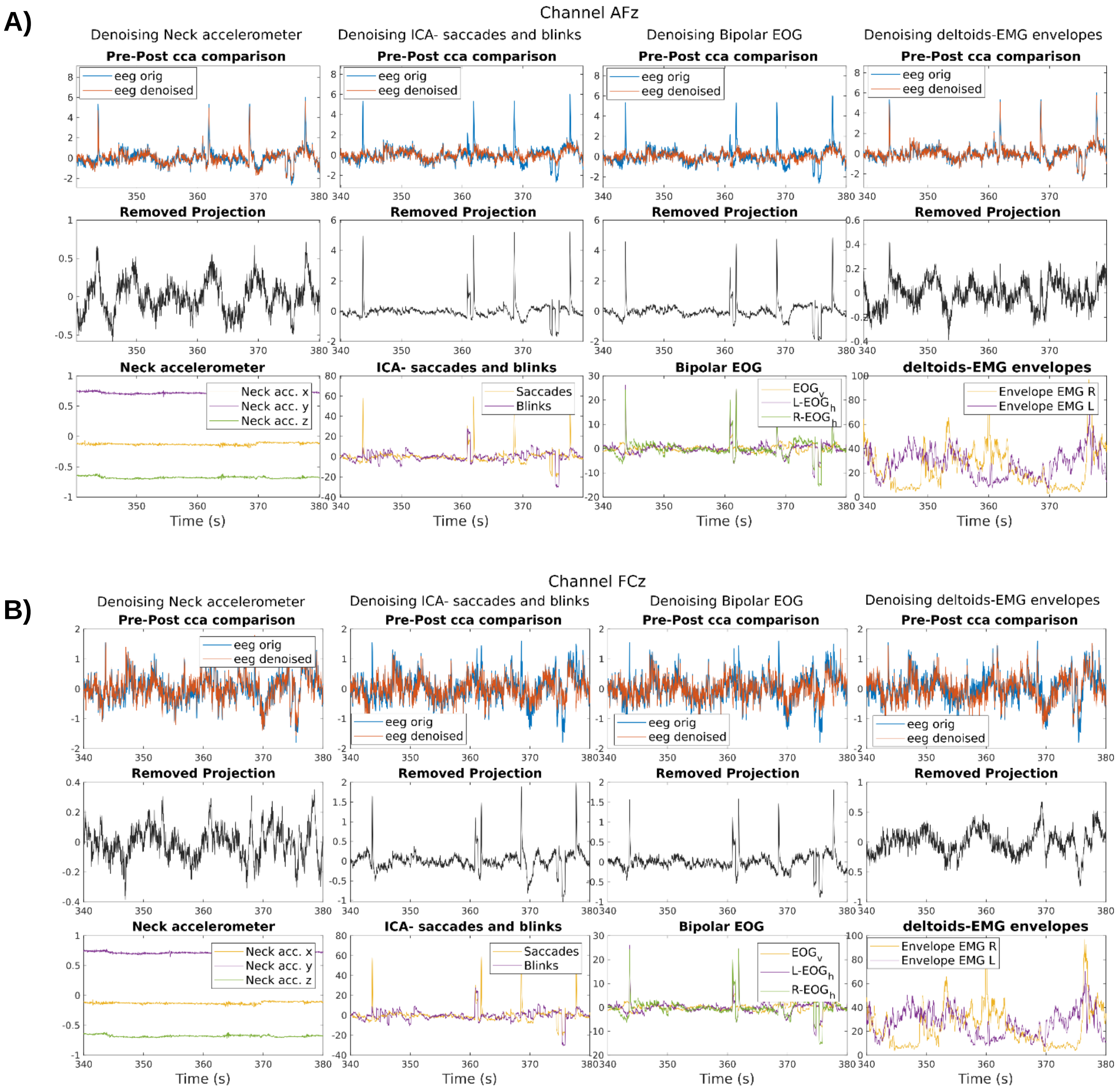

Supplementary Figure 1. Effect of CCA denoising procedure on two selected electrodes (panel A for AFz and panel B for FCz) for some representative confounding variables (columns): neck accelerometers, ICA-detected blinks and saccades, bipolar-EOG derivations, deltoids-EMG envelopes. The figure shows 40-seconds results of a representative subjects Top-row: EEG channel before and noise removal. Middle row: cca-projection to be removed; Bottom-row: confounding variable to be removed.

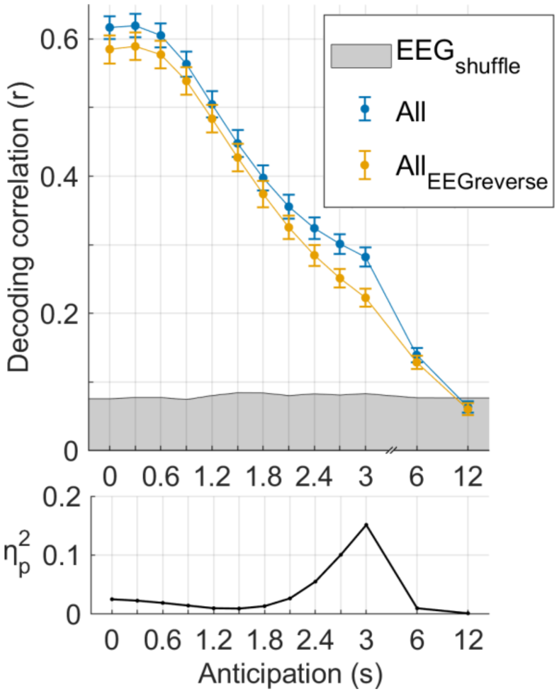

Supplementary Figure 2. The steering signal was predicted by combining EEG, EMG, and accelerometric data in the same LLR decoder (All). To verify that the EEG signal carries unique predictive information not available in the EMG and accelerometric data, we run the same decoding analyses after time-reversing the EEG information (AllEEGreverse). The contrast between the two indicates that the EEG signal carries significant anticipatory information not available in the EMG and accelerometric data between up to 3s of anticipation. The LLR window was set at 1.5s. Error-bars indicate the SD across participants. The grey shaded area indicates the baseline prediction correlation (95th percentile of the null distribution). The plot on the bottom reports the effect-size for All > AllEEGreverse.
